# Nitric oxide signals are interlinked with calcium signals in normal pancreatic stellate cells upon oxidative stress and inflammation

**DOI:** 10.1098/rsob.160149

**Published:** 2016-08-03

**Authors:** Monika A. Jakubowska, Pawel E. Ferdek, Oleg V. Gerasimenko, Julia V. Gerasimenko, Ole H. Petersen

**Affiliations:** 1 Medical Research Council Group, School of Biosciences, Cardiff University, Cardiff CF10 3AX, Wales, UK; 2 Systems Immunity Research Institute, Cardiff University, Cardiff CF14 4XN, Wales, UK

**Keywords:** calcium, inflammation, nitric oxide, pancreas, stellate cell

## Abstract

The mammalian diffuse stellate cell system comprises retinoid-storing cells capable of remarkable transformations from a quiescent to an activated myofibroblast-like phenotype. Activated pancreatic stellate cells (PSCs) attract attention owing to the pivotal role they play in development of tissue fibrosis in chronic pancreatitis and pancreatic cancer. However, little is known about the actual role of PSCs in the normal pancreas. These enigmatic cells have recently been shown to respond to physiological stimuli in a manner that is markedly different from their neighbouring pancreatic acinar cells (PACs). Here, we demonstrate the capacity of PSCs to generate nitric oxide (NO), a free radical messenger mediating, for example, inflammation and vasodilatation. We show that production of cytosolic NO in PSCs is unambiguously related to cytosolic Ca^2+^ signals. Only stimuli that evoke Ca^2+^ signals in the PSCs elicit consequent NO generation. We provide fresh evidence for the striking difference between signalling pathways in PSCs and adjacent PACs, because PSCs, in contrast to PACs, generate substantial Ca^2+^-mediated and NOS-dependent NO signals. We also show that inhibition of NO generation protects both PSCs and PACs from necrosis. Our results highlight the interplay between Ca^2+^ and NO signalling pathways in cell–cell communication, and also identify a potential therapeutic target for anti-inflammatory therapies.

## 1. Background

Mammalian stellate cells (Latin *stella—*star) are retinoid-storing cells woven into the tissue of various organs [1] including the liver, pancreas, kidney, spleen, lung and vocal folds. Stellate cells are capable of transformations from a quiescent to an activated myofibroblast-like phenotype [2]. Activated stellate cells have attracted attention owing to the pivotal role they play in pathological fibrosis: they overproduce extracellular matrix proteins to repair the chronic stress-induced injuries in the tissue [1–3]. Nevertheless, the initial pathophysiological role of stellate cells—prior to activation—remains enigmatic. Here, we studied the primary signalling events, evoked by either oxidative stress or proinflammatory mediators, in stellate cells (PSCs) and neighbouring acinar cells (PACs) in the normal mouse pancreas, and identified a link between calcium and nitric oxide signalling pathways in PSCs.

In the normal pancreas acetylcholine (ACh) or cholecystokinin (CCK) evoke Ca^2+^ signals regulating the processes of enzyme release from zymogen granules deposited in the apical parts of PACs [4–10]. However, under pathological conditions (e.g. bile reflux into the pancreatic duct, high-fat diet together with excessive alcohol intake), Ca^2+^ signals become abnormally large and this elicits premature activation of enzymes within PACs and subsequent necrosis [8], followed by sterile (non-microbial) inflammation leading to acute pancreatitis (AP) [11,12].

Reactive oxygen/nitrogen species (ROS/RNS), such as NO, are highly chemically active radical and non-radical molecules that initiate and propagate reactions of oxidative stress, and thus act as second messengers in various inflammatory processes [13–16]. Both endogenous and exogenous ROS can modulate store-operated Ca^2+^ entry (SOCE) [17–20] and release [21]. Excessive Ca^2+^ influx into PACs, together with the sustained elevation of the cytosolic calcium ion concentration ([Ca^2+^]_C_) [22–24], underlies the mechanism of AP [25], and store-operated Ca^2+^ entry channels are therefore potential therapeutic targets [24,26–28]. Nevertheless, the roles of ROS/RNS (including NO) in the (patho)physiology of PSCs remain unexplored. Generation of NO has not yet been reported in PSCs, although a plausible link between PSC activation and NO has been established: in cultured rat PSCs, expression of nitric oxide synthase 2 (NOS2) is increased after stimulation with pathogen-associated molecular patterns (PAMP) that activate Toll-like receptors (TLR) of innate immunity [29]. TLRs also mediate responses to damage-associated molecular patterns (DAMP) released from injured tissues (e.g. the necrotizing pancreas) [12].

The proinflammatory mediator bradykinin (BK) induces NO production in vascular endothelial cells [30,31]. BK was recently shown to elicit Ca^2+^ signals in PSCs [32], at pathophysiologically relevant concentrations [33], and this was linked to AP via specific action on PSCs through bradykinin receptor B2 [33]. So far, it is unknown whether BK elicits NO generation in PSCs and, if so, what the role of this process might be. Bile acids (BA), employed extensively in cellular [11,22,34,35] and animal [16,36–38] studies of the pancreas, induce abnormal Ca^2+^ signals [34] that cause necrosis and AP [25]. In the light of the inflammatory background of AP, it seems likely that BK and BA would evoke not only Ca^2+^ signals, but also NO signals with the potential for crosstalk between the two signalling pathways.

In order to explore such interactions, we inhibited either Ca^2+^ or NO signal generation in PSCs. Caffeine is known to reduce Ca^2+^ signals via inhibition of inositol 1,4,5-triphosphate receptors in PACs [39,40]; what is more, a caffeine-dependent decline in severity of pancreatic injury was recently demonstrated in three animal models of AP [41]. Thus, caffeine was used here to test whether the blockade of BA-elicited Ca^2+^ responses might attenuate NO signals. The latter were also blocked pharmacologically by inhibitors of enzymatic NO synthesis (NOS inhibitors), widely used in therapy of various inflammatory diseases [42], including AP [43,44]. Nevertheless, so far, the actual outcome of NOS inhibition in AP remains unclear. The aim of this study was to investigate the role of normal PSCs in the initial signalling events upon proinflammatory stimulation. We report here that PSCs, in contrast to PACs, generate substantial Ca^2+^-mediated and NOS-dependent NO signals, and that inhibition of NO generation protects both PSCs and PACs from necrosis.

## 2. Methods

*Animals*: C57BL6/J mice (Charles Rivers). *Antibodies:* Alexa Fluor 488 goat anti-mouse, Alexa Fluor 635 goat anti-rabbit (Thermo Fisher Scientific); mouse anti-BDKRB2 (Santa Cruz); rabbit anti-NOS2 (Merck). *Cell culture:* human pancreatic stellate cell line, SteCM complete stellate cell medium (ScienCell). *Chemicals*: aminoguanidine (AG), bradykinin, l-NAME (Tocris); PBS, ProLong Diamond Antifade Mountant with DAPI (Thermo Fisher Scientific); other chemicals were obtained from Sigma. *Fluorescent dyes:* DAF-2 (Santa Cruz); DAF-FM, Fluo-4, Fura-2, Hoechst 33342, propidium iodide (Thermo Fisher Scientific).

### 2.1. Isolation of pancreatic lobules

Six- to eight-week-old male mice were sacrificed by cervical dislocation, the pancreases were dissected and the lobules were immediately isolated by collagenase digestion. Briefly, the pancreas was injected intraductally with NaHEPES-based collagenase solution and incubated (5–6 min, 37°C) to allow partial digestion of the tissue.

### 2.2. Primary human pancreatic stellate cell line

hPSCs were cultured (up to the fifth passage) at 37°C, 5% CO_2_, in complete stellate cell medium and split once a week.

### 2.3. Cytosolic calcium or nitric oxide measurements

Unless otherwise indicated, NaHEPES-based media, containing (mM): NaCl, 140; KCl, 4.7; HEPES, 10; MgCl_2_, 1; glucose, 10; and pyruvate, 1, were supplemented with 1 mM Ca^2+^ for calcium measurements and with 1 mM Ca^2+^ together with 0.5 mM l-Arg for nitric oxide recordings. For Ca^2+^ measurements, the lobules were loaded with 10 µM Fluo-4 (1 h, 30°C), and hPSC with 1 µM Fluo-4 (30 min, 37°C). For NO measurements, the lobules were loaded with 20 µM DAF-2 or DAF-FM (1 h, 30°C), and hPSC with 0.1 µM DAF-2 or DAF-FM (1 h, 37°C). The lobules were transferred to a flow chamber and allowed to adhere to the glass surface; and for hPSC imaging, the coverslips with growing cells were used for flow chamber assembly. Experiments were performed in continuous perfusion with extracellular buffer-based solution; and the cells were visualized using a TCS SP5 II two-photon confocal microscope (Leica) with a 63 × 1.2 NA water objective. Fluo-4 or DAF dyes were excited with a 488 nm Ar laser, at 1–4% power, and emitted light was collected in the three-dimensional recording mode at 495–580 nm. The speed of recordings was approximately one image per 10 s, and varied dependent on thickness of the samples (up to 15 µm, *z*-axis resolution 1 µm). Images were captured at 512 × 512, and series of images were recorded at 256 × 256 pixel resolution, respectively, and analysed using Leica software. In order to reconstruct the three-dimensional signal, the individual signals from z-stacks were cropped, and the maximal projection was applied. Fluorescence signals were plotted as *F*/*F*_0_, where *F*_0_ was an averaged signal from first ten baseline images, and normalized as previously described [45].

### 2.4. Simultaneous cytosolic calcium and nitric oxide measurements

For simultaneous Ca^2+^ and NO measurements, the lobules were loaded with 10 µM Fura-2 and 10 µM DAF-2 (1 h, 30°C). After the loading, the lobules were transferred to the chamber, perfused and visualized as described above. Fura-2 fluorescence was excited with 355 nm and 405 nm lasers, at 8% and 16% power, respectively; and emitted light was collected in the three-dimensional recording mode at 500–600 nm; DAF-2 fluorescence was excited and collected as described above.

### 2.5. Measurements of necrosis level in the lobules

The lobules were treated with 5 mM cholate, 5 mM taurocholate or 0.2 mM TLC-S challenge (2 h, room temperature), and in some experiments, 0.6 mM l-NAME was present. The lobular PSCs were visualized using Fluo-4 (10 µM, 2 h); the lobules were co-stained with Hoechst 33342 (32 µM, 30 min), and dead cells were identified by PI staining (1.5 µM, 15 min) as described [33]. The cells were visualized, using the confocal microscope with a 63 × 1.2 NA water objective. Fluo-4, Hoechst 33342 and PI were excited with 488 nm Ar (1%), 355 nm diode (10%) and 543 nm HeNe laser (10%), respectively; and corresponding emissions were collected at 505–535, 415–485 and 615–720 nm. The fluorescence signal was collected sequentially between frames in the three-dimensional mode from 20 µm thick lobules and 512 × 512 pixel resolution. Five pictures of independent lobules were taken in each of four experimental replicates (*n* = 20), and live (PI-negative) and dead (PI-positive) cells were counted.

### 2.6. Immunohistochemistry

Unless otherwise indicated, the procedure was performed at room temperature, and double distilled water (ddH_2_O) was used for preparation of all solutions. 0.1% Tween 20 was used as a washing buffer and 1% BSA in PBS with 0.1% Tween 20 was a blocking buffer. Mouse pancreatic tissue samples were fixed in formalin, embedded in paraffin and cut into 4 µm sections. The sections were heated in a dry oven (30 min, 65°C), then deparaffinized in xylene (2 × 10 min) and graded ethanol, and then incubated in 50 mM NH_4_Cl (20 min). Antigen retrieval was achieved by autoclaving (20 min, 120°C) the samples in TAE buffer (pH 8.1), followed by slow cooling to room temperature (30 min). Permeabilization was performed in 0.4% Triton X-100 (10 min). In order to quench autofluorescence, the sections were incubated in 0.2% Sudan black B [46]. The sections were then transferred to a humid chamber, and blocking of non-specific binding sites was performed (1 h), followed by incubation with primary anti-BDKRB2 and anti-NOS2 Abs (0.5 µg ml^−1^) for 1 h at room temperature, and then overnight at 4°C. The negative controls were incubated in blocking solution with no primary Abs. The following day, the sections were incubated (1 h) with goat anti-rabbit secondary Ab (4 µg ml^−1^), washed, and then incubated (1 h) with goat anti-mouse secondary Ab (4 µg ml^−1^). The sections were embedded in antifade mounting medium with DAPI, and imaged immediately using the confocal microscope (excitation wavelengths: 355, 488 and 633 nm). The slides were stored at 4°C.

### 2.7. Statistics

The quantitative results were expressed as means ± s.d. or s.e.m. (see the text for details). Statistical analysis was performed using the Student's *t*-test or ANOVA, and the significance threshold was set at 0.05.

## 3. Results

### 3.1. Oxidative stress elicits (patho)physiological calcium and nitric oxide signals

**Figure 1.**
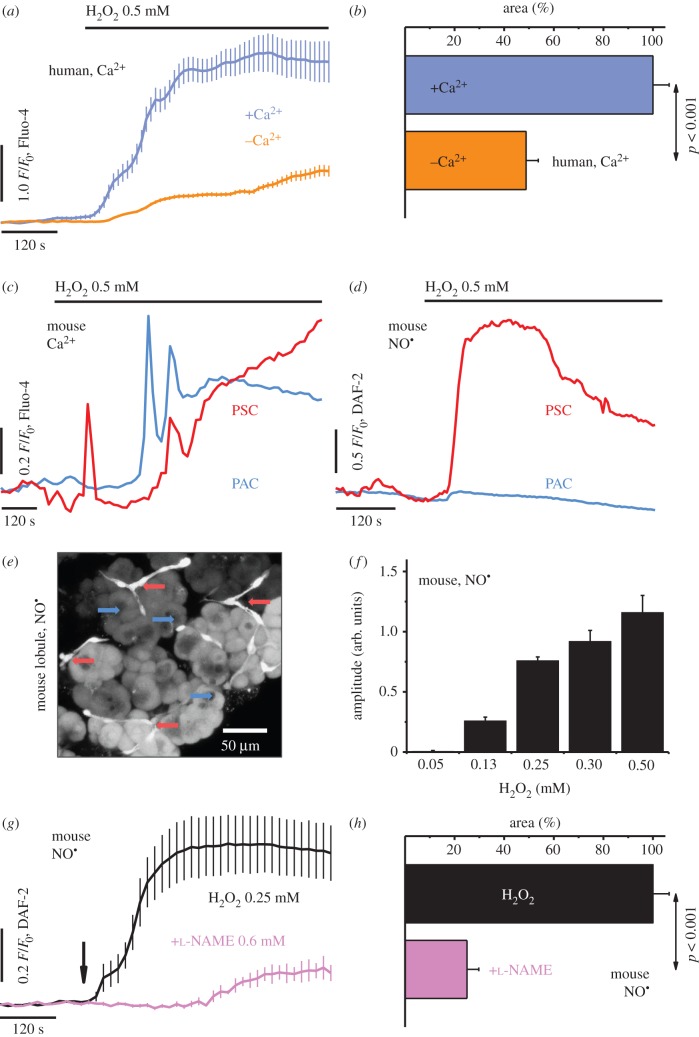
Oxidative stress evokes cytosolic calcium and nitric oxide signals in the pancreatic cells. (*a*) Average traces (mean ± s.e.m.) of cytosolic Ca^2+^ responses to 0.5 mM H_2_O_2_ in hPSCs, in the presence of 1.0 mM Ca^2+^ (blue, *n* = 38) or the absence of Ca^2+^ (orange, *n* = 35). (*b*) Bar chart (mean ± s.e.m.) comparing the areas under the response curves to 0.5 mM H_2_O_2_ in the presence of 1.0 mM Ca^2+^ (blue, *n* = 38) or the absence of Ca^2+^ (orange, *n* = 35)—summary of (*a*). (*c*) Typical cytosolic Ca^2+^ responses elicited by 0.5 mM H_2_O_2_ in PSCs (red, *n* = 6) and PACs (blue, *n* = 6) in pancreatic lobules. (*d*) Typical cytosolic NO responses elicited by 0.5 mM H_2_O_2_ in PSCs (red, *n* = 5) and PACs (blue, *n* = 8) in pancreatic lobules. See also electronic supplementary material, video S1 and figure S1. (*e*) Photomicrograph of the lobules loaded with fluorescent NO probe DAF-FM, upon stimulation with 1 mM H_2_O_2_. Arrows: PSCs, red; PACs, blue. Scale bar, 50 µm. (*f*) Bar chart (mean ± s.e.m.) comparing amplitudes of cytosolic NO responses in PSCs, elicited by 0.05–0.5 mM H_2_O_2_ in the lobules (*n* ≥ 5). (*g*) Average traces (mean ± s.e.m.) of cytosolic NO responses to 0.25 mM H_2_O_2_ (arrow) in the absence (black, *n* = 13) or the presence of 0.6 mM l-NAME (pink, *n* = 10), in PSCs in the lobules. (*h*) Bar chart (mean ± s.e.m.) comparing the areas under the response curves of PSCs to 0.25 mM H_2_O_2_ in the absence (black, *n* = 13) or the presence of 0.6 mM l-NAME (pink, *n* = 10)—the summary of (*g*).

Hydrogen peroxide (H_2_O_2_) was used as an initiator of oxidative stress in human pancreatic stellate cells (hPSCs; figure 1*a–b*) and mouse pancreatic tissue lobules (figure 1*c*–*h*). In hPSCs, a sustained increase in [Ca^2+^]_C_ was evoked by 0.5 mM H_2_O_2_ (blue), which was markedly (*p* < 0.001) attenuated by removal of external Ca^2+^ (orange; figure 1*a*–*b*). In the lobules, oxidative stress elicited rises in [Ca^2+^]_C_ in both PSCs (red) and PACs (blue; figure 1*c*), although cytosolic NO signals (figure 1*d*; see also electronic supplementary material, figure S1 and video S1) were limited spatially to PSCs (red), manifest as a sharp increase and sustained plateau phase. Three-dimensional reconstruction of the DAF-FM fluorescence signal collected from the 1 mM H_2_O_2_-stimulated lobules confirmed the increase in [NO]_C_ *in situ* in PSCs (cells of well-defined projections, red arrows), but not in adjacent PACs (cells that form the acini, blue arrows; figure 1*e*). The amplitudes of the H_2_O_2_-elicited increases in cytosolic NO in the PSCs varied, depending on the peroxide concentration (figure 1*f* ). The development of NO responses was modulated by the NOS inhibitor N(ω)-nitro-l-arginine methyl ester (l-NAME; figure 1*g*–*h*), so that, at a concentration of 0.6 mM (pink trace and bar), it attenuated the 0.25 mM H_2_O_2_-evoked (black trace and bar) NO signals (figure 1*g*), reducing significantly (*p* < 0.001) the area under the response curve (figure 1*h*).

### 3.2. The proinflammatory mediator bradykinin evokes simultaneous calcium and nitric oxide signals in stellate cells

**Figure 2.**
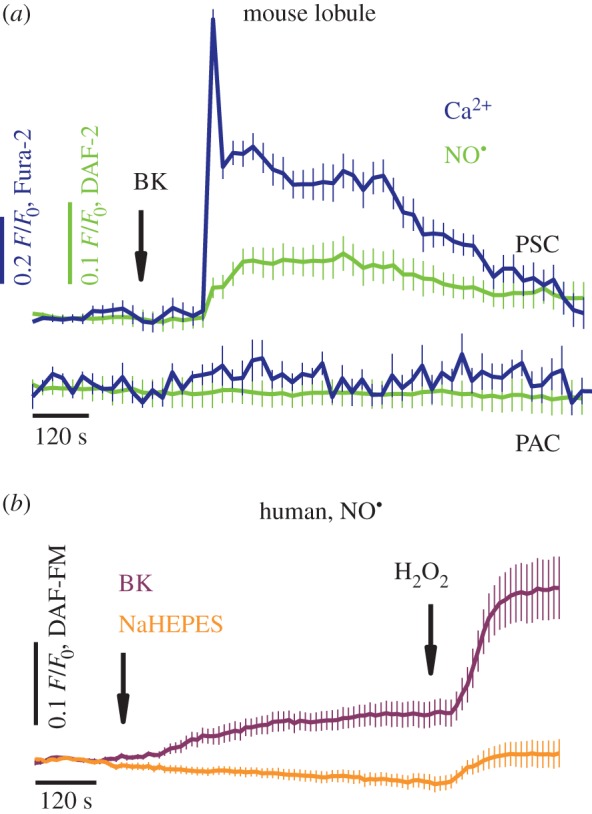
Bradykinin elicits simultaneous cytosolic calcium and nitric oxide responses in stellate cells. (*a*) Average traces (mean ± s.e.m.) of cytosolic Ca^2+^ and NO responses to 20 nM BK (arrow), recorded in pancreatic lobules. The lobules were loaded with Fura-2 and DAF-2 fluorescent probes, and Ca^2+^ (navy blue) and NO responses (green) were registered simultaneously in PSCs (*n* = 12; top curves); no responses were detected in PACs (*n* = 6; bottom curves). (*b*) Average traces (mean ± s.e.m.) of cytosolic NO responses to 1 µM BK, and then to 0.5 mM H_2_O_2_ recorded in human PSCs (purple, *n* = 24); the control cells received placebo and H_2_O_2_ treatment (orange, *n* = 24).

Double loading of the lobules—with Ca^2+^- and NO-sensing fluorescent indicators—revealed simultaneous development of both Ca^2+^ and NO signals: upon stimulation of the lobules with 20 nM BK (figure 2*a*), a rapid increase in [Ca^2+^]_C_, with an initial peak and subsequent plateau phase (navy blue), and a sustained increase in cellular NO [NO]_C_ (green) were observed solely in PSCs, with no Ca^2+^/NO signals detected in PACs (figure 2*a*). In hPSCs, a sustained increase in [NO]_C_ was evoked by 1 µM BK (figure 2*b*), and additional stimulation of the cells with 0.5 mM H_2_O_2_ brought a further rise in [NO]_C_.

### 3.3. Bile-acid-elicited calcium and nitric oxide signals have different profiles in acinar and stellate cells

**Figure 3.**
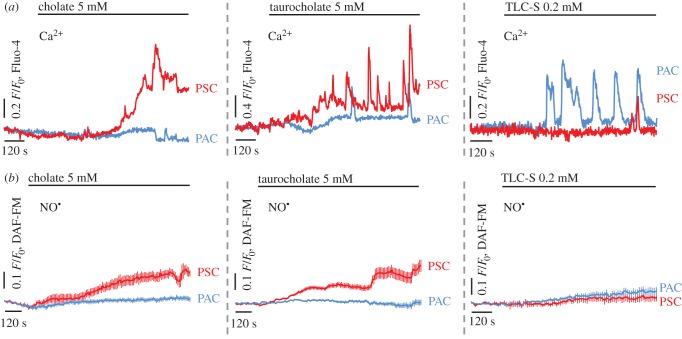
Bile acids evoke toxic calcium overload and nitric oxide signals in the lobules. (*a*) Typical cytosolic Ca^2+^ responses elicited by 5 mM cholate (left), 5 mM taurocholate (centre) and 0.2 mM TLC-S (right) in PSCs (red; *n* = 5, *n* = 6, *n* = 4) and PACs (blue; *n* = 12, *n* = 7, *n* = 19). (*b*) Typical cytosolic NO responses elicited by 5 mM cholate (left), 5 mM taurocholate (central) and 0.2 mM TLC-S (right) in PSCs (red; *n* = 8, shown also in figure 4*a* and electronic supplementary material, figure S2*a*; *n* = 16, shown also in figure 4*b*; *n* = 5) and PACs (blue; *n* = 8, *n* = 6, *n* = 8). See also electronic supplementary material, figure S2.

Robust [Ca^2+^]_C_ elevations in the lobules (figure 3*a*) were evoked by the natural bile sodium salts: 5 mM cholate (left), 5 mM taurocholate (middle) and 0.2 mM taurolithocholic acid 3-sulfate (TLC-S; right). The pattern of the responses was markedly different in adjacent PACs and PSCs: cholate and taurocholate-elicited increases in [Ca^2+^]_C_ almost exclusively in PSCs (red), with robust Ca^2+^ signals in cholate- and oscillatory Ca^2+^ signals in taurocholate-stimulated PSCs, whereas only very modest responses—single spikes—were detected in PACs (blue). In contrast, stimulation with TLC-S evoked Ca^2+^ signals with an oscillatory pattern in PACs (blue), and almost no detectable responses in PSCs (red). NO levels were shown to rise in PSCs (red) upon stimulation with cholate (figure 3*b*, left; see also electronic supplementary material, figure S2) and taurocholate (figure 3*b*, middle), but not with TLC-S (figure 3*b*, right); no detectable NO signals accompanied the Ca^2+^ signals in PACs (blue). 20 mM caffeine blocked the development of cholate-evoked NO signals in both lobular PSCs and hPSCs (electronic supplementary material, figure S2).

### 3.4. Nitric oxide synthase inhibitors modulate bile-acid-evoked nitric oxide signals

**Figure 4.**
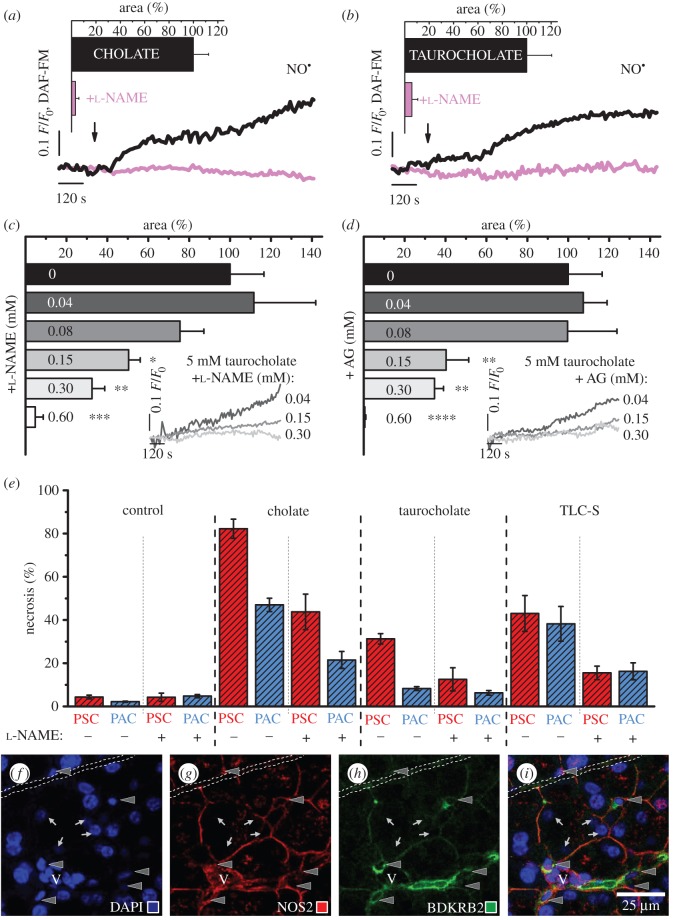
Nitric oxide synthase inhibitors diminish cytosolic nitric oxide signals and protect from necrosis. (*a*) Typical cytosolic NO responses evoked by 5 mM cholate in the absence (black, *n* = 8) or the presence of 0.6 mM l-NAME (pink, *n* = 9). Inset: bar chart (mean ± s.e.m.) comparing the areas under the response curves. (*b*) Typical cytosolic NO responses evoked by 5 mM taurocholate in the absence (black, *n* = 16) or the presence of 0.6 mM l-NAME (pink, *n* = 8). Inset: bar chart (mean ± s.e.m.) comparing the areas under the response curves (showed also in figure 4*c*). (*c*) Bar chart (mean ± s.e.m.) comparing the areas under the response curves of PSCs to 5 mM taurocholate, in the presence of 0.04–0.6 mM l-NAME (*n* ≥ 3). Inset: typical cytosolic NO responses of PSCs to 5 mM taurocholate in the presence of 0.04 (dark grey), 0.15 (grey) and 0.3 mM l-NAME (light grey); the responses are presented from the moment of taurocholate and NOS inhibitor administration. (*d*) Bar chart (mean ± s.e.m.) comparing the areas under the response curves of PSCs to 5 mM taurocholate, in the presence of 0.04–0.6 mM AG (*n* ≥ 3). Inset: typical cytosolic NO responses of PSCs to 5 mM taurocholate in the presence of 0.04 (dark grey), 0.15 (grey) and 0.3 mM AG (light grey); the responses are presented as in figure 4*c*. (*e*) Bar chart (mean ± s.d.) comparing levels of necrosis in the lobules under different experimental conditions. The lobules were treated with 5 mM cholate, 5 mM taurocholate or 0.2 mM TLC-S, the presence of 0.6 mM l-NAME is indicated with +; NaHEPES-treated lobules served as control. PSCs (dashed red) and PACs (dashed blue) were counted after triple staining of the lobules with fluorescent probes: Fluo-4, Hoechst 33342 and PI (four replicates, *n* = 20). See also electronic supplementary material, figure S3. ( *f*–*i*) Co-immunolocalization of the BDKRB2 and the NOS2 in mouse pancreas. ( *f* ) DAPI, (*g*) Ab anti-NOS2, (*h*) Ab anti-BDKRB2, (*i*) overlaid ( *f*–*h*). Scale bar, 25 µm. Arrowheads, PSCs; arrows, PACs; V, blood vessel; dash line, pancreatic duct. See also electronic supplementary material, figure S4.

Pharmacological NOS inhibitors, the non-specific l-NAME and the irreversible inhibitor of NOS2 aminoguanidine (AG), reduced BA-evoked NO responses in the lobules (figure 4*a*–*d*). The developments of 5 mM cholate- (figure 4*a*) and 5 mM taurocholate-elicited (figure 4*b*) responses (black traces and bars) were significantly reduced in the presence of 0.6 mM l-NAME (pink traces and bars; figure 4*a*–*b*). The aforementioned reductions in taurocholate-elicited NO generation were dependent on l-NAME concentration (figure 4*c*; *p* < 0.05*, *p* < 0.01** and *p* < 0.001***), as well as AG concentration (figure 4*d*; *p* < 0.01** and *p* < 0.0001****), as shown in the bar charts (0.04–0.6 mM inhibitor; figure 4*c*–*d*) and the representative traces in the insets (0.04, 0.15 and 0.3 mM inhibitor; figure 4*c*–*d*).

### 3.5. Blockade of nitric oxide production reduces necrosis in the pancreatic lobules

The role of pharmacological blockade of NO production was analysed after 2 h incubation of the lobules with 5 mM cholate, 5 mM taurocholate or 0.2 mM TLC-S (figure 4*e*; see also electronic supplementary material, figure S3), and l-NAME was used as NOS inhibitor. Because the levels of necrosis in the lobules that received 0.6 mM l-NAME (and no BA) treatment did not change in comparison with NaHEPES controls (less than 5%), l-NAME at this concentration was tested as a protective agent against the BA challenge. Lobular PSCs (dashed red) were substantially more susceptible to cholate or taurocholate than PACs (dashed blue): the PSCs/PACs necrosis ratios were approximately 83/47 (1.8) in cholate- and approximately 31/8 (3.8) in taurocholate-treated lobules. The presence of l-NAME, however, reduced necrosis by almost 50%, irrespective of cell type, reaching approximately 48/22 (2.2) for the cholate- and approximately 13/6 (1.3) for taurocholate-challenged lobules, respectively. TLC-S induced necrosis at similar levels in both pancreatic cell types: the PSCs/PACs necrosis was approximately 43/38 (1.1), and in l-NAME-protected lobules approximately 16/16 (1.0), similarly the cholate- or taurocholate-treated lobule necrosis decreased by almost 50%.

### 3.6. Source of nitric oxide signals in pancreatic stellate cells

Co-immunolocalization of NOS2 and BK receptor B2 (BDKRB2) in the pancreas was assessed in formalin-fixed paraffin-embedded mouse tissue sections (figure 4*f*–*i*; see also electronic supplementary material, figure S4), and DAPI was used to stain the nuclei (PSCs: elongated, indicated with arrowheads; PACs: large round, indicated with arrows; figure 4*f*). The PSCs were localized in the interacinar spaces, encircling the base of adjacent acini with fine cytoplasmic processes. The processes, as well as the areas that surround PSCs nuclei, were visualized using anti-NOS2 (figure 4*g*, red) and anti-BDKRB2 (figure 4*h*, green) antibodies (Ab). The areas of co-immunolocalization (figure 4*i*, hybrid yellow to orange) were identified as PSCs. The transmitted light image along with the high-resolution NOS2 staining images are shown in electronic supplementary material, figure S4.

## 4. Discussion and conclusion

It has previously been shown that cytosolic calcium signals can be elicited in cultured [32] and in normal (lobular) [33] pancreatic stellate cells. This study now reveals that these signals are interlinked with cytosolic nitric oxide signals.

We show that NO signals are evoked in PSCs upon induction of oxidative stress (figure 1) or application of inflammatory mediators (figures 2–4). Oxidative stress originates from the imbalance between the production and neutralization of ROS/RNS [13,47], and is implicated in the mechanisms of numerous inflammatory diseases [15,16,20], including AP [11,16,19]. However, for pancreatitis, there remains the ‘chicken or the egg’ question regarding the roles of ROS/RNS in the development of inflammation. Here, we show that H_2_O_2_ at concentrations that are (patho)physiologically relevant [47,48] evoke large Ca^2+^ signals with an oscillatory pattern in both PACs and PSCs (figure 1*a*–*c*). Importantly, we demonstrate that it is only in the PSCs that these Ca^2+^ signals are accompanied by detectable NO signals (figure 1*d*–*h*).

Spatial separations of BK- and ACh- or CCK-elicited Ca^2+^ signals in pancreatic lobules have previously been reported. Whereas ACh and CCK evoke Ca^2+^ signals in PACs, which control normal acinar secretion [49], BK-evoked Ca^2+^ signals are entirely confined to PSCs [33]. BK-elicited Ca^2+^ signalling events in PSCs are mediated via BDKRB2 [33] and pharmacological blockade of this receptor with the B2 antagonist WIN64338 protected lobular PACs from the necrosis evoked by alcohol/fatty acid or bile acids [33], indicating a possible paracrine interaction between PSCs and PACs. Here, we show that BK elicits simultaneous Ca^2+^ and NO signals in PSCs (figure 2*a*, upper curves), but fails to evoke responses in adjacent PACs (figure 2*a*, lower curves). Because large Ca^2+^ signals in PSCs are not accompanied by Ca^2+^ signals in PACs, and vice versa, it may be NO that mediates paracrine communication between stellate cells and other cell types in the pancreas.

Bile acids are natural compounds of the bile that facilitate enzymatic hydrolysis of lipids in the process of digestion [50]. In the case of gallstone-induced bile reflux into the pancreatic duct, bile acids in high concentrations will get in direct contact with pancreatic cells. Bile acids evoke large abnormal Ca^2+^ signals in PACs [22,34,35], followed by intracellular enzyme activation, PAC necrosis [25], autodigestion of the pancreas and finally pancreatitis [11,12,25]. Cytoplasmic and mitochondrial Ca^2+^ signals, elicited in isolated PACs by TLC-S, impair ATP synthesis and induce ROS production [11]. Nevertheless, TLC-S has little effect on [Ca^2+^]_C_ in PSCs (figure 3*a*), and no detectable role in NO signalling in these cells (figure 3*b*). In contrast, robust Ca^2+^ signals elicited in PSCs by cholate and taurocholate (figure 3*a*) are accompanied by NO signals (figures 3*b* and 4*a*–*d*). A plausible explanation for the diverse sensitivity of pancreatic cells to bile acids might be the different pattern of bile-acid-transporting proteins in PSCs and PACs.

Our results demonstrating that even substantial Ca^2+^ signals generated in PACs fail to elicit detectable NO signals are in agreement with a previous study in which the non-enzymatic NO signal generation was explored in isolated PACs [45]. In that study, even supramaximal ACh concentrations failed to evoke detectable NO signals in more than 70% of intact PACs.

The potential benefit of blocking NO generation in the therapy of various diseases, including AP, remains controversial [15]: several studies show beneficial effects of NOS inhibitors in the therapy of cancer [51], arthritis [52] and pancreatitis [43,44], whereas others demonstrate that blockade of NO production aggravates liver injury [53], and exacerbates inflammation in the kidney [54]. Here, we show that l-NAME (blocker of NOS 1–3; figure 4*a*–*c*) and AG (irreversible inhibitor of NOS2; figure 4*d*) significantly reduce bile-acid-evoked NO signals in PSCs. Furthermore, we demonstrate that there is substantially more necrosis in PSCs than in PACs in cholate- or taurocholate-challenged lobules (figure 4*e*), which correlates with the presence of NO signals together with large Ca^2+^ signals in PSCs upon stimulation with these BAs (figure 3). Interestingly, the levels of necrosis in TLC-S-stressed lobules are comparable for both type of pancreatic cells (figure 4*e*)—possibly owing to the lack of detectable NO signals in PSCs (figure 3) that could exacerbate necrosis. The pharmacological inhibitor l-NAME significantly reduces necrosis in BA-challenged lobules, irrespective of bile type and cell type (figure 4*e*). In cholate- or taurocholate-stressed lobules the protective effect of l-NAME (blockade of NO signals ameliorates necrosis) is more prominent in the PSCs than in the PACs, which do not have detectable cytosolic NO signals. The protection of PACs might be the result of, for example, paracrine communication, PSCs–PACs, via intercellular messengers (plausibly NO) released by PSCs.

Expression of the NO-forming enzymes, NOS 1–3, in the exocrine pancreas has recently been confirmed using state-of-the-art immunohistological methods [55]. The authors of the study, however, did not refer directly to PSCs, although they report the presence of ‘cells of the morphology of ductal cells’ in chronic pancreatitis (CP) tissue specimens [55]. These cells could well have been PSCs, which are known to induce fibrosis in CP [2,56]. TLR-mediated expression of NOS2 has been detected in isolated PSCs upon stimulation with PAMP [29]. Importantly, TLRs are also activated upon stimulation with DAMP—endogenous molecules released from injured cells [12] (e.g. autodigested PACs). Thus, DAMP-exposed and then NOS2-expressing PSCs might be present in the pancreatic tissue. Here, using the Ab anti-BDKRB2 (figure 4*h*; green), we identify PSCs in mouse pancreatic tissue specimens: the cells localize in the interacinar spaces, encircling the base of adjacent acini with fine cytoplasmic processes [57]. This is in line with other immunohistological studies on PSCs in the milieu of the tissue [1,57]. The pattern of anti-NOS2 Ab staining (figure 4*g*; red) resembles the network of discrete stellate-shaped cells and their cytoplasmic processes [57]. The staining of BDKRB2 and NOS2 overlay, yielding yellow to orange colour (figure 4*i*). The strong staining of PSC processes might be explained by the results of a study on polarized epithelial cells [58] in which NOS2 was shown to be compartmentalized in the submembrane areas (here: near the membrane surrounding the nuclear area and covering the very fine cytoplasmic processes). Such submembrane localization of NOS2 would ensure precise vertical delivery (across the cell membrane) of the second messenger NO to the target [58,59].

NO generated in PSCs may not only exert an effect on PACs, but also reach endothelial cells in peri-acinar capillaries. As pointed out recently [60], the PSCs are strategically localized in a niche between acinar cells and peri-acinar capillaries, and, in the intact pancreas *in vivo*, NO generated in PSCs could well have vascular effects.

In conclusion, our results reveal interplay between NO and Ca^2+^ *in situ* in PSCs, induced by stress related to inflammation (figures 1–2) and disease (figures 3–4). PSCs might then resemble a sensor that downstream the stress signals by means of at least two second messengers.

## Supplementary Material

Supplementary Material

## Data accessibility

Figures S1–S4 supporting this article have been uploaded as part of the electronic supplementary material. Datasets for this article have been collected and processed using Leica LAS AF software (https://leica-microsystems.com/products/microscope-software) needed to open the original files. Other supporting files will be made available in the Research section via: https://www.cardiff.ac.uk/people/view/108900-gerasimenko-oleg, and Dryad Digital Repository (electronic supplementary material, video S1): http://dx.doi.org/10.5061/dryad.4rb47.

## References

[1] Zhao L, Burt AD 2007 The diffuse stellate cell system. J. Mol. Histol. 38, 53–64. (doi:10.1007/s10735-007-9078-5) 17294244 10.1007/s10735-007-9078-5

[2] Erkan M et al. 2012 StellaTUM: current consensus and discussion on pancreatic stellate cell research. Gut 61, 172–178. (doi:10.1136/gutjnl-2011-301220) 22115911 10.1136/gutjnl-2011-301220 PMC3245897

[3] Sherman MH et al. 2014 Vitamin D receptor-mediated stromal reprogramming suppresses pancreatitis and enhances pancreatic cancer therapy. Cell 159, 80–93. (doi:10.1016/j.cell.2014.08.007) 25259922 10.1016/j.cell.2014.08.007 PMC4177038

[4] Hegyi P, Petersen OH 2013 The exocrine pancreas: the acinar-ductal tango in physiology and pathophysiology. Rev. Physiol. Biochem. Pharmacol. 165, 1–30. (doi:10.1007/112_2013_14) 23881310 10.1007/112_2013_14

[5] Tepikin AV, Voronina SG, Gallacher DV, Petersen OH 1992 Acetylcholine-evoked increase in the cytoplasmic Ca^2+^ concentration and Ca^2+^ extrusion measured simultaneously in single mouse pancreatic acinar cells. J. Biol. Chem. 267, 3569–3572. 1310974

[6] Thorn P, Lawrie AM, Smith PM, Gallacher DV, Petersen OH 1993 Local and global cytosolic Ca^2+^ oscillations in exocrine cells evoked by agonists and inositol trisphosphate. Cell 74, 661–668. (doi:10.1016/0092-8674(93)90513-P) 8395347 10.1016/0092-8674(93)90513-p

[7] Gerasimenko OV, Gerasimenko JV, Belan PV, Petersen OH 1996 Inositol trisphosphate and cyclic ADP-ribose-mediated release of Ca^2+^ from single isolated pancreatic zymogen granules. Cell 84, 473–480. (doi:10.1016/S0092-8674(00)81292-1) 8608601 10.1016/s0092-8674(00)81292-1

[8] Raraty M, Ward J, Erdemli G, Vaillant C, Neoptolemos JP, Sutton R, Petersen OH 2000 Calcium-dependent enzyme activation and vacuole formation in the apical granular region of pancreatic acinar cells. Proc. Natl Acad. Sci. USA 97, 13 126–13 131. (doi:10.1073/pnas.97.24.13126) 10.1073/pnas.97.24.13126 PMC27189 11087863

[9] Matthews EK, Petersen OH, Williams JA 1973 Pancreatic acinar cells: acetylcholine-induced membrane depolarization, calcium efflux and amylase release. J. Physiol. 234, 689–701. (doi:10.1113/jphysiol.1973.sp010367) 4764435 10.1113/jphysiol.1973.sp010367 PMC1350694

[10] Case RM, Clausen T 1973 The relationship between calcium exchange and enzyme secretion in the isolated rat pancreas. J. Physiol. 235, 75–102. (doi:10.1113/jphysiol.1973.sp010379) 4778143 10.1113/jphysiol.1973.sp010379 PMC1350734

[11] Booth DM et al. 2011 Reactive oxygen species induced by bile acid induced apoptosis and protect against necrosis in pancreatic acinar cells. Gastroenterology 140, 2116–2125. (doi:10.1053/j.gastro.2011.02.054) 21354148 10.1053/j.gastro.2011.02.054

[12] Hoque R, Malik AF, Gorelick F, Mehal WZ 2012 Sterile Inflammatory response in acute pancreatitis. Pancreas 41, 353–357. (doi:10.1097/MPA.0b013e3182321500) 22415665 10.1097/MPA.0b013e3182321500 PMC3306133

[13] Halliwell B 1991 Reactive oxygen species in living systems: source, biochemistry, and role in human disease. Am. J. Med. 91, S14–S22. (doi:10.1016/0002-9343(91)90279-7) 10.1016/0002-9343(91)90279-7 1928205

[14] Kerwin JJF, Lancaster JJR, Feldman PL 1995 Nitric oxide: a new paradigm for second messengers. J. Med. Chem. 38, 4343–4362. (doi:10.1021/jm00022a001) 7473563 10.1021/jm00022a001

[15] Pacher P, Beckman JS, Liaudet L 2007 Nitric oxide and peroxynitrite in health and disease. Physiol. Rev. 87, 315–424. (doi:10.1152/physrev.00029.2006) 17237348 10.1152/physrev.00029.2006 PMC2248324

[16] Lerch MM, Gorelick FS 2013 Models of acute and chronic pancreatitis. Gastroenterology 144, 1180–1193. (doi:10.1053/j.gastro.2012.12.043) 23622127 10.1053/j.gastro.2012.12.043

[17] Bogeski I, Klich T, Niemeyer BA 2012 ROS and SOCE: recent advances and controversies in the regulation of STIM and Orai. J. Physiol. 590, 4193–4200. (doi:10.1113/jphysiol.2012.230565) 22615429 10.1113/jphysiol.2012.230565 PMC3473278

[18] Bruce JIE, Elliott AC 2007 Oxidant-impaired intracellular Ca^2+^ signaling in pancreatic acinar cells: role of the plasma membrane Ca^2+^-ATPase. Am. J. Physiol. Cell Physiol. 293, C938–C950. (doi:10.1152/ajpcell.00582.2006) 17494627 10.1152/ajpcell.00582.2006

[19] Baggaley EM, Elliott AC, Bruce JIE 2008 Oxidant-induced inhibition of the plasma membrane Ca^2+^-ATPase in pancreatic acinar cells: role of the mitochondria. Am. J. Physiol. Cell Physiol. 295, C1247–C1260. (doi:10.1152/ajpcell.00083.2008) 18787078 10.1152/ajpcell.00083.2008 PMC2584981

[20] Trebak M, Ginnan R, Singer HA, Jourd'heuil D 2010 Interplay between calcium and reactive oxygen/nitrogen species: an essential paradigm for vascular smooth muscle signaling. Antioxid. Redox Signal. 12, 657–674. (doi:10.1089/ars.2009.2842) 19719386 10.1089/ars.2009.2842 PMC2861541

[21] Ivanova H, Vervliet T, Missiaen L, Parys JB, De Smedt H, Bultynck G 2014 Inositol 1,4,5-trisphosphate receptor-isoform diversity in cell death and survival. Biochim. Biophys. Acta 1843, 2164–2183. (doi:10.1016/j.bbamcr.2014.03.007) 24642269 10.1016/j.bbamcr.2014.03.007

[22] Voronina SG, Gryshchenko OV, Gerasimenko OV, Green AK, Petersen OH, Tepikin AV 2005 Bile acids induce a cationic current, depolarizing pancreatic acinar cells and increasing the intracellular Na^+^ concentration. J. Biol. Chem. 280, 1764–1770. (doi:10.1074/jbc.M410230200) 15536077 10.1074/jbc.M410230200

[23] Criddle DN, Raraty MGT, Neoptolemos JP, Tepikin AV, Petersen OH, Sutton R 2004 Ethanol toxicity in pancreatic acinar cells: mediation by nonoxidative fatty acid metabolites. Proc. Natl Acad. Sci. USA 101, 10 738–10 743. (doi:10.1073/pnas.0403431101) 10.1073/pnas.0403431101 PMC490004 15247419

[24] Gerasimenko JV et al. 2013 Ca^2+^ release-activated Ca^2+^ channel blockade as a potential tool in antipancreatitis therapy. Proc. Natl Acad. Sci. USA 110, 13 186–13 191. (doi:10.1073/pnas.1300910110) 10.1073/pnas.1300910110 PMC3740877 23878235

[25] Petersen OH, Sutton R 2006 Ca^2+^ signalling and pancreatitis: effects of alcohol, bile and coffee. Trends Pharmacol. Sci. 27, 113–120. (doi:10.1016/j.tips.2005.12.006) 16406087 10.1016/j.tips.2005.12.006

[26] Wen L et al. 2015 Inhibitors of ORAI1 prevent cytosolic calcium-associated injury of human pancreatic acinar cells and acute pancreatitis in 3 mouse models. Gastroenterology 149, 481–492. (doi:10.1053/j.gastro.2015.04.015) 25917787 10.1053/j.gastro.2015.04.015 PMC4556985

[27] Parekh AB, Putney JW Jr 2005 Store-operated calcium channels. Physiol. Rev. 85, 757–810. (doi:10.1152/physrev.00057.2003) 15788710 10.1152/physrev.00057.2003

[28] Hegyi P 2016 Blockade of calcium entry provides a therapeutic window in acute pancreatitis. J. Physiol. 594, 257 (doi:10.1113/jp271710) 26767885 10.1113/JP271710 PMC4713745

[29] Masamune A, Kikuta K, Watanabe T, Satoh K, Satoh A, Shimosegawa T 2008 Pancreatic stellate cells express toll-like receptors. J. Gastroenterol. 43, 352–362. (doi:10.1007/s00535-008-2162-0) 18592153 10.1007/s00535-008-2162-0

[30] Palmer RMJ, Ferrgie AG, Moncada S 1987 Nitric oxide release accounts for the biological activity of endothelium-derived relaxing factor. Nature 327, 524–526. (doi:10.1038/327524a0) 3495737 10.1038/327524a0

[31] Palmer RMJ, Aston DS, Moncada S 1988 Vascular endothelial cells synthesize nitric oxide from L-arginine. Nature 333, 664–666. (doi:10.1038/333664a0) 3131684 10.1038/333664a0

[32] Won JH, Zhang Y, Ji B, Logsdon CD, Yule DI 2011 Phenotypic changes in mouse pancreatic stellate cell Ca^2+^ signaling events following activation in culture and in a disease model of pancreatitis. Mol. Biol. Cell 22, 421–436. (doi:10.1091/mbc.E10-10-0807) 21148289 10.1091/mbc.e10-10-0807 PMC3031471

[33] Gryshchenko O, Gerasimenko JV, Gerasimenko OV, Petersen OH 2016 Ca^2+^ signals mediated by bradykinin type 2 receptors in normal pancreatic stellate cells can be inhibited by specific Ca^2+^ channel blockade. J. Physiol. 594, 281–293. (doi:10.1113/JP271468) 26442817 10.1113/JP271468 PMC4713750

[34] Voronina S, Longbottom R, Sutton R, Petersen OH, Tepikin A 2002 Bile acids induce calcium signals in mouse pancreatic acinar cells: implications for bile-induced pancreatic pathology. J. Physiol. 540, 49–55. (doi:10.1113/jphysiol.2002.017525) 11927668 10.1113/jphysiol.2002.017525 PMC2290202

[35] Gerasimenko JV, Flowerdew SE, Voronina SG, Sukhomlin TK, Tepikin AV, Petersen OH, Gerasimenko OV 2006 Bile acids induce Ca^2+^ release from both the endoplasmic reticulum and acidic intracellular calcium stores through activation of inositol trisphosphate receptors and ryanodine receptors. J. Biol. Chem. 281, 40 154–40 163. (doi:10.1074/jbc.M606402200) 10.1074/jbc.M606402200 17074764

[36] Perides G, van Acker GJD, Laukkarinen JM, Steer ML 2010 Experimental acute biliary pancreatitis induced by retrograde infusion of bile acids into the mouse pancreatic duct. Nat. Protoc. 5, 335–341. (doi:10.1038/nprot.2009.243) 20134432 10.1038/nprot.2009.243

[37] Laukkarinen JM, van Acker GJD, Weiss ER, Steer ML, Perides G 2007 A mouse model of acute biliary pancreatitis induced by retrograde pancreatic duct infusion of Na-taurocholate. Gut 56, 1590–1598. (doi:10.1136/gut.2007.124230) 17591621 10.1136/gut.2007.124230 PMC2095649

[38] Muili KA et al. 2013 Bile acids induce pancreatic acinar cell injury and pancreatitis by activating calcineurin. J. Biol. Chem. 288, 570–580. (doi:10.1074/jbc.M112.428896) 23148215 10.1074/jbc.M112.428896 PMC3537054

[39] Wakui M, Osipchuk YV, Petersen OH 1990 Receptor-activated cytoplasmic Ca^2+^ spiking mediated by inositol trisphosphate is due to Ca^2+^-induced Ca^2+^ release. Cell 63, 1025–1032. (doi:10.1016/0092-8674(90)90505-9) 1701691 10.1016/0092-8674(90)90505-9

[40] Toescu EC, O'Neill SC, Petersen OH, Eisner DA 1992 Caffeine inhibits the agonist-evoked cytosolic Ca^2+^ signal in mouse pancreatic acinar cells by blocking inositol trisphosphate production. J. Biol. Chem. 267, 23 467–23 470. 1429689

[41] Huang W et al. 2015 Caffeine protects against experimental acute pancreatitis by inhibition of inositol 1,4,5-trisphosphate receptor-mediated Ca^2+^ release. Gut. (doi:10.1136/gutjnl-2015-309363) 10.1136/gutjnl-2015-309363 PMC5284483 26642860

[42] Laszlo F, Evans SM, Whittle BJ 1995 Aminoguanidine inhibits both constitutive and inducible nitric oxide synthase isoforms in rat intestinal microvasculature *in vivo*. Eur. J. Pharmacol. 272, 169–175. (doi:10.1016/0014-2999(94)00637-M) 7536162 10.1016/0014-2999(94)00637-m

[43] Hegyi P, Rakonczay Z Jr 2011 The role of nitric oxide in the physiology and pathophysiology of the exocrine pancreas. Antioxid. Redox Signal. 15, 2723–2741. (doi:10.1089/ars.2011.4063) 21777142 10.1089/ars.2011.4063

[44] Camargo EA et al. 2014 Inhibition of inducible nitric oxide synthase-derived nitric oxide as a therapeutical target for acute pancreatitis induced by secretory phospholipase A2. Eur. J. Pain. 18, 691–700. (doi:10.1002/j.1532-2149.2013.00414.x) 24166730 10.1002/j.1532-2149.2013.00414.x

[45] Chvanov M, Gerasimenko OV, Petersen OH, Tepikin AV 2006 Calcium-dependent release of NO from intracellular s-nitrosothiols. EMBO J. 25, 3024–3032. (doi:10.1038/sj.emboj.7601207) 16810320 10.1038/sj.emboj.7601207 PMC1500983

[46] Sun Y, Yu H, Zheng D, Cao Q, Wang Y, Harris D, Wang Y 2011 Sudan black B reduces autofluorescence in murine renal tissue. Arch. Pathol. Lab. Med. 135, 1335–1342. (doi:10.5858/arpa.2010-0549-OA) 21970489 10.5858/arpa.2010-0549-OA

[47] Droge W 2002 Free radicals in the physiological control of cell function. Physiol. Rev. 82, 47–95. (doi:10.1152/physrev.00018.2001) 11773609 10.1152/physrev.00018.2001

[48] Coombes E, Jiang J, Chu XP, Inoue K, Seeds J, Branigan D, Simon RP, Xiong ZG 2011 Pathophysiologically relevant levels of hydrogen peroxide induce glutamate-independent neurodegeneration that involves activation of transient receptor potential melastatin 7 channels. Antioxid. Redox Signal. 14, 1815–1827. (doi:10.1089/ars.2010.3549) 20812867 10.1089/ars.2010.3549 PMC3078500

[49] Petersen OH, Tepikin AV 2008 Polarized calcium signaling in exocrine gland cells. Annu. Rev. Physiol. 70, 273–299. (doi:10.1146/annurev.physiol.70.113006.100618) 17850212 10.1146/annurev.physiol.70.113006.100618

[50] Hofmann AF 1999 Bile acids: the good, the bad, and the ugly. News. Physiol. Sci. 14, 24–29. 11390813 10.1152/physiologyonline.1999.14.1.24

[51] Belgorosky D, Langle Y, Prack Mc Cormick B, Colombo L, Sandes E, Eijan AM 2014 Inhibition of nitric oxide is a good therapeutic target for bladder tumors that express iNOS. Nitric Oxide 36, 11–18. (doi:10.1016/j.niox.2013.10.010) 24211345 10.1016/j.niox.2013.10.010

[52] McCartney-Francis N, Allen JB, Mizel DE, Albina JE, Xie QW, Nathan CF, Wahl SM 1993 Suppression of arthritis by an inhibitor of nitric oxide synthase. J. Exp. Med. 178, 749–754. (doi:10.1084/jem.178.2.749) 7688035 10.1084/jem.178.2.749 PMC2191124

[53] Wang Y, Lawson JA, Jaeschke H 1998 Differential effect of 2-aminoethyl-isothiourea, an inhibitor of the inducible nitric oxide synthase, on microvascular blood flow and organ injury in models of hepatic ischemia-reperfusion and endotoxemia. Shock 10, 20–25. (doi:10.1097/00024382-199807000-00004) 9688086 10.1097/00024382-199807000-00004

[54] Rangan GK, Wang Y, Harris DC 2001 Pharmacologic modulators of nitric oxide exacerbate tubulointerstitial inflammation in proteinuric rats. J. Am. Soc. Nephrol. 12, 1696–1705. 11461942 10.1681/ASN.V1281696

[55] Buchwalow I, Schnekenburger J, Tiemann K, Samoilova V, Bankfalvi A, Poremba C, Schleicher C, Neumann J, Boecker W 2013 L-Arginine-NO-cGMP signalling pathway in pancreatitis. Sci. Rep. 3, 1899 (doi:10.1038/srep01899) 23712581 10.1038/srep01899 PMC3664897

[56] Bachem MG et al. 1998 Identification, culture, and characterization of pancreatic stellate cells in rats and humans. Gastroenterology 115, 421–432. (doi:10.1016/S0016-5085(98)70209-4) 9679048 10.1016/s0016-5085(98)70209-4

[57] Apte MV, Haber PS, Applegate TL, Norton ID, McCaughan GW, Korsten MA, Pirola RC, Wilson JS 1998 Periacinar stellate shaped cells in rat pancreas: identification, isolation, and culture. Gut 43, 128–133. (doi:10.1136/gut.43.1.128) 9771417 10.1136/gut.43.1.128 PMC1727174

[58] Glynne PA, Darling KE, Picot J, Evans TJ 2002 Epithelial inducible nitric-oxide synthase is an apical EBP50-binding protein that directs vectorial nitric oxide output. J. Biol. Chem. 277, 33 132–33 138. (doi:10.1074/jbc.M205764200) 10.1074/jbc.M205764200 12080081

[59] Parekh AB 2011 Decoding cytosolic Ca^2+^ oscillations. Trends Biochem. Sci. 36, 78–87. (doi:10.1016/j.tibs.2010.07.013) 20810284 10.1016/j.tibs.2010.07.013

[60] Gryshchenko O, Gerasimenko JV, Gerasimenko OV, Petersen OH 2016 Calcium signalling in pancreatic stellate cells: mechanisms and potential roles. Cell Calcium 59, 140–144. (doi:10.1016/j.ceca.2016.02.003) 26960936 10.1016/j.ceca.2016.02.003

