# Supplementary material for: Nitric oxide signals are interlinked with calcium signals in normal pancreatic stellate cells upon oxidative stress and inflammation
Source: Open Biol. 2016 Aug 3;6(8):160149. doi: 10.1098/rsob.160149 (PMC5008014; doi:10.1098/rsob.160149)
Supplement: Supplementary Material [file rsob160149supp1.pdf]

Supplementary Material

Jakubowska et al. 2016, "Nitric Oxide Signals are Interlinked with Calcium Signals in Normal Pancreatic Stellate Cells upon Oxidative Stress and Inflammation"

Fig. S1

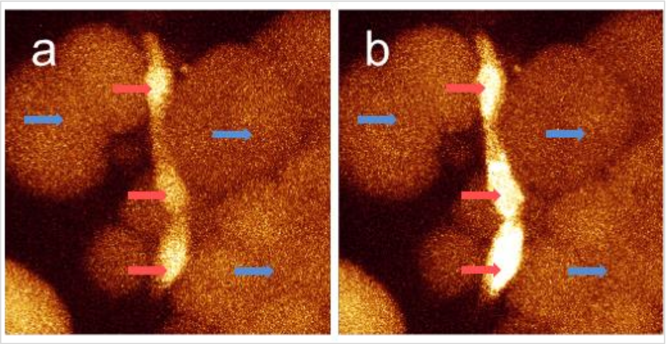

Fig. S1 Oxidative stress induces increase in cytosolic nitric oxide in stellate cells - images from Movie S1.

a The pancreatic tissue lobule loaded with DAF-FM fluorescent NO indicator. Arrows: PSCs red, PACs blue.

b The same lobule stimulated with 0.5 mM H_2O_2.

Fig. S2

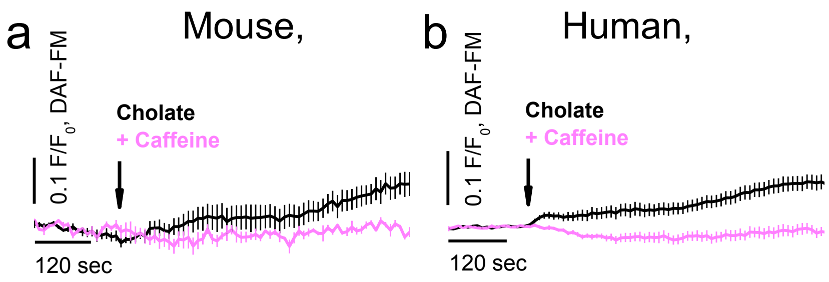

Fig. S2 Inhibitor of 1,4,5-triphosphate receptors caffeine attenuates development of cytosolic nitric oxide signals.

a Average traces (mean±SEM) of cytosolic NO responses to 5 mM cholate in the absence (black, n=8) and the presence of 20 mM caffeine (pink, n=5), exerted in the lobular PSCs.

b Average traces (mean±SEM) of cytosolic NO responses to 1 mM cholate in the absence (black, n=35) and the presence of 20 mM caffeine (pink, n=12), exerted in hPSCs.

Fig. S3

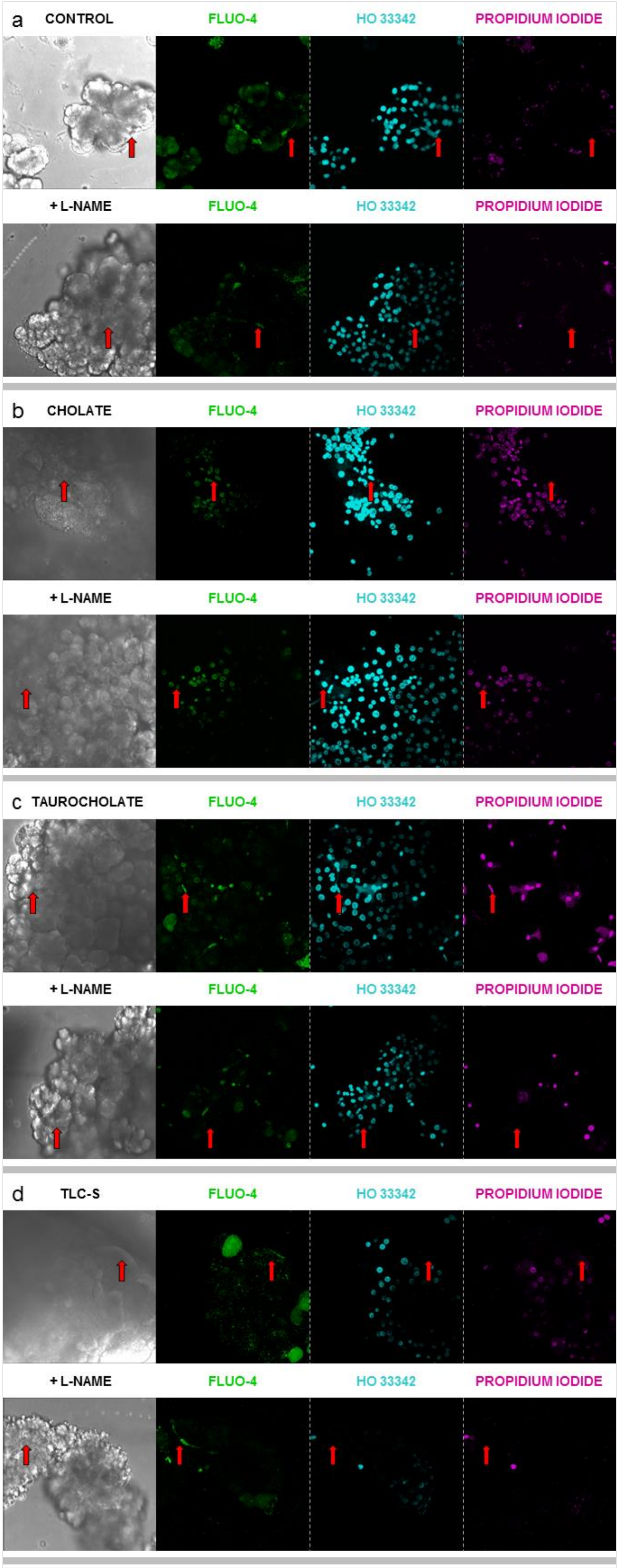

a CONTROL
FLUO-4
HO 33342
PROPIDIUM IODIDE
+ L-NAME
FLUO-4
HO 33342
PROPIDIUM IODIDE
b CHOLATE
FLUO-4
HO 33342
PROPIDIUM IODIDE
+ L-NAME
FLUO-4
HO 33342
PROPIDIUM IODIDE
c TAUROCHOLATE
FLUO-4
HO 33342
PROPIDIUM IODIDE
+ L-NAME
FLUO-4
HO 33342
PROPIDIUM IODIDE
d TLC-S
FLUO-4
HO 33342
PROPIDIUM IODIDE
+ L-NAME
FLUO-4
HO 33342
PROPIDIUM IODIDE

Fig. S3 Photomicrographs comparing levels of necrosis in the lobules under different experimental conditions - graphical summary of Fig. 4e.

The lobules were triple loaded with fluorescent dyes Fluo-4 (green, left), Hoechst 33342 (cyan, middle) and PI (magenta, right), and followed the bile acid-challenge, and some of them were treated in the presence of 0.6 mM L-NAME (indicated with +). Arrows: PSCs. Representative transmitted light and fluorescent images are shown in the panels: **a** The untreated (control) lobules. **b** The 5 mM cholate-challenged lobules. **c** The 5 mM taurocholate-challenged lobules. **d** The 0.2 mM TLC-S-challenged lobules.

Fig. S4

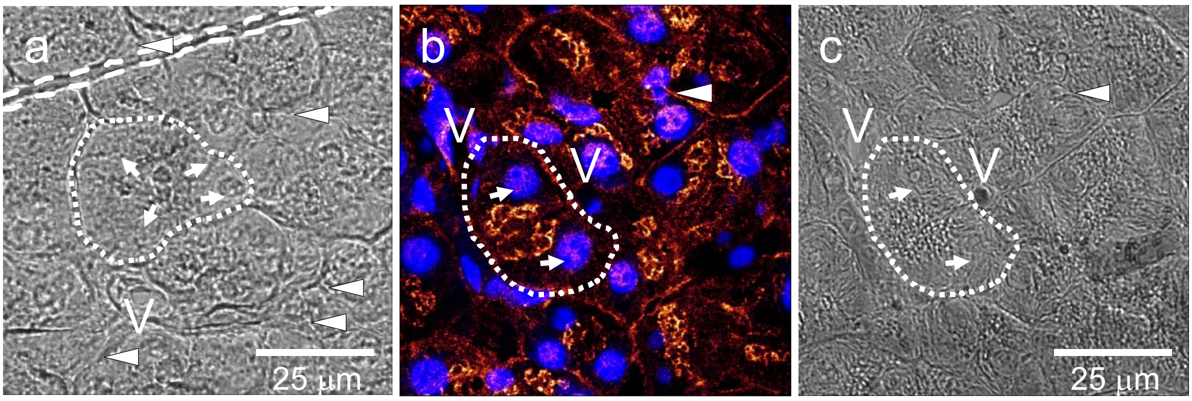

Fig. S4 Mouse exocrine pancreas - paraffin-embedded tissue.

a Transmitted light image of **Figs. 4f-i.** Arrowheads - PSCs, arrows - PACs, V - blood vessels, dashed line - pancreatic duct, dotted line - acinus. Scale bar: 25 µm.

b High-resolution immunolocalisation of NOS2 in mouse pancreas. Nuclei stained with DAPI (blue); NOS2 (yellow). Arrowheads - PSCs, arrows - PACs, V - blood vessel, dotted line - acinus.

c Transmitted light image of **b**. Scale bar: 25 µm.
